# Effect of Nuclear Stiffness on Cell Mechanics and Migration of Human Breast Cancer Cells

**DOI:** 10.3389/fcell.2020.00393

**Published:** 2020-05-29

**Authors:** Tony Fischer, Alexander Hayn, Claudia Tanja Mierke

**Affiliations:** Biological Physics Division, Peter Debye Institute of Soft Matter Physics, Faculty of Physics and Earth Sciences, Leipzig University, Leipzig, Germany

**Keywords:** cell mechanics, deformability, stiffness, viscoelasticity, cancer cells, invasion, nuclear mechanics, 3D collagen matrices

## Abstract

The migration and invasion of cancer cells through 3D confined extracellular matrices is coupled to cell mechanics and the mechanics of the extracellular matrix. Cell mechanics is mainly determined by both the mechanics of the largest organelle in the cell, the nucleus, and the cytoskeletal architecture of the cell. Hence, cytoskeletal and nuclear mechanics are the major contributors to cell mechanics. Among other factors, steric hindrances of the extracellular matrix confinement are supposed to affect nuclear mechanics and thus also influence cell mechanics. Therefore, we propose that the percentage of invasive cells and their invasion depths into loose and dense 3D extracellular matrices is regulated by both nuclear and cytoskeletal mechanics. In order to investigate the effect of both nuclear and cytoskeletal mechanics on the overall cell mechanics, we firstly altered nuclear mechanics by the chromatin de-condensing reagent Trichostatin A (TSA) and secondly altered cytoskeletal mechanics by addition of actin polymerization inhibitor Latrunculin A and the myosin inhibitor Blebbistatin. In fact, we found that TSA-treated MDA-MB-231 human breast cancer cells increased their invasion depth in dense 3D extracellular matrices, whereas the invasion depths in loose matrices were decreased. Similarly, the invasion depths of TSA-treated MCF-7 human breast cancer cells in dense matrices were significantly increased compared to loose matrices, where the invasion depths were decreased. These results are also valid in the presence of a matrix-metalloproteinase inhibitor GM6001. Using atomic force microscopy (AFM), we found that the nuclear stiffnesses of both MDA-MB-231 and MCF-7 breast cancer cells were pronouncedly higher than their cytoskeletal stiffness, whereas the stiffness of the nucleus of human mammary epithelial cells was decreased compared to their cytoskeleton. TSA treatment reduced cytoskeletal and nuclear stiffness of MCF-7 cells, as expected. However, a softening of the nucleus by TSA treatment may induce a stiffening of the cytoskeleton of MDA-MB-231 cells and subsequently an apparent stiffening of the nucleus. Inhibiting actin polymerization using Latrunculin A revealed a softer nucleus of MDA-MB-231 cells under TSA treatment. This indicates that the actin-dependent cytoskeletal stiffness seems to be influenced by the TSA-induced nuclear stiffness changes. Finally, the combined treatment with TSA and Latrunculin A further justifies the hypothesis of apparent nuclear stiffening, indicating that cytoskeletal mechanics seem to be regulated by nuclear mechanics.

## Introduction

Cell migration and invasion are inherently coupled to cell mechanics and matrix environmental mechanics (Mierke et al., 2008, 2011a,b, 2018; Baker and Chen, 2012; Fruleux and Hawkins, 2016; Dietrich et al., 2018; Mierke, 2019a), or in other words their elastic properties, such as stiffness. Cell mechanics are intertwined with matrix mechanics, since the environmental mechanics can alter cell mechanics and in turn cells can remodel the surrounding matrix environment by exerting forces on it (Wolf et al., 2009, 2013; Mierke, 2011; Mierke et al., 2011b, 2017; Fischer et al., 2017; Kunschmann et al., 2019). Another way for matrix remodeling is through matrix-metalloproteinases that are secreted by cells to degrade steric hindrances for cell migration (Mierke et al., 2011a; Yu et al., 2012; Wolf et al., 2013; Davidson et al., 2014; Harada et al., 2014; Denais et al., 2016; Das et al., 2017, 2019). All of these features contribute to the regulation of cellular motility in 3D confined extracellular matrices. There is still the general hypothesis that cancer cell mechanics contributes “universally” to the 3D migration, as cancer cells with an increased invasive capacity have displayed increased 3D motility and are more deformable and hence softer than cancer cells with a decreased invasive capacity (Guck et al., 2005; Cross et al., 2007; Remmerbach et al., 2009; Fritsch et al., 2010; Runge et al., 2014; Lekka, 2016; Meinhövel et al., 2018). In contrast, it has been hypothesized that a “universal” mechanism for all cancer cell types (Jonietz, 2012; Alibert et al., 2017) or even all cell types (Mierke, 2019b) is probably not suitable for migration into 3D extracellular matrix confinements due to the multiple biochemical and genetic differences among the vastly different cell types. Instead, there may also be other mechanisms employed that explain why even the opposite behavior can be observed, which means that even the stiffer cancer cells or stiffer fibroblasts, migrate more efficiently into 3D extracellular matrix confinements (Mierke et al., 2008; Mierke, 2011; Kunschmann et al., 2017, 2019).

The mechanical properties of cells can be determined with many biophysical techniques, such as optical stretching, atomic force microscopy, magnetic tweezer, traction force microscopy or 3D matrix displacement analysis (Guck et al., 2005; Mierke et al., 2008, 2011a; Lekka, 2016; Fischer et al., 2017; Kunschmann et al., 2017, 2019) and therefore may even lead to contradictory results. In fact, the differences of the findings have been attributed to different cell mechanics probing techniques and different culture conditions or different cell types or cell lines. However, not everything can be explained by technical differences, and a central question remained whether these techniques usually examine bulk properties of rather anisotropic cells. Unlike other soft matter materials, a cell contains organelles and contains a rather non-homogenously structured cytoskeleton. More precisely, the largest cellular organelle, the cell nucleus can play an important role in this scenario. In turn, external mechanical forces transmitted through the cell can directly alter the shape, its position inside the cells (Lele et al., 2018) and even the function of the nucleus, such as the altered expression of genes (Mack et al., 2001; Brooks et al., 2002). Moreover, these proteins can then interfere with multiple processes of the entire cell (Dahl Kris et al., 2008; Fedorchak et al., 2014), ultimately disrupting the internal remodeling of the nucleoskeletal structure and chromatin structure (Dahl Kris et al., 2008; Dahl and Kalinowski, 2011; Guolla et al., 2012; Lele et al., 2018), which in turn leads to additional changes in gene transcription (Booth-Gauthier et al., 2012; Iyer et al., 2012). It is not yet understood exactly how nuclei react to physical signals and whether this is related to their inherent mechanical properties. However, it has been revealed that the chromatin structure undergoes alterations upon matrix mechanics changes (Le et al., 2016; Miroshnikova et al., 2017; Rabineau et al., 2018). In fact, there are two distinct mechanical response regimes of the nucleus, such as small and large mechanical perturbations that are handled by chromatin and lamins, respectively (Stephens et al., 2017, 2018a,b, 2019).

Therefore, it is clearly necessary to determine how the nucleus deforms and remodels in response to force in order to explain obvious differences in the stiffness (deformability) of cells. There is a strictly balanced regulation of the nuclear shape and consequently of nuclear mechanics, such as nuclear stiffness in migrating and invading cells. In detail, the nuclear prestress regulates the nuclear shape and the cytoskeletal organization preserves the nuclear shape by external mechanical stress (Rowat et al., 2006; Mazumder and Shivashankar, 2010; Dahl and Kalinowski, 2011). There are some basic questions to be answered, e.g., whether the observed mechanical anisotropy is a widespread phenomenon of different cells or not. More specifically, when the nuclear anisotropy is present in cells, how can it be regulated during the migration of cells through the 3D extracellular matrix, which fully surrounds these migrating cancer cells? Can cytoskeletal structures, such as actin or myosin, and nuclear components, such as the condensation state of chromatin, affect cellular anisotropy and subsequently 3D migration?

Firstly, to investigate whether the nuclear deformability regulates the migratory capacity of cancer cells in 3D extracellular matrix confinements, we performed 3D collagen matrix invasion assays in the presence and absence of TSA. In the absence of TSA, the MDA-MB-231 cells invaded deeper into dense matrices compared to loose matrices. In contrast, MCF-7 cells invaded less into dense matrices compared to loose matrices. Finally, MDA-MB-231 cells were more invasive compared to MCF-7 cells in both types of matrices. In the presence of TSA, the percentage of invasive cells were slightly or pronouncedly increased for the two cell types and both matrices. Moreover, the invasion depths of both cell types were significantly decreased in loose matrices, but significantly increased in dense matrices.

Secondly, to explore whether the nuclear stiffness (deformability) can regulate the migratory capacity of cancer cells in 3D extracellular matrix confinements, we analyzed the effect of TSA on nuclear stiffness. More precisely, we found that the nuclear stiffness of MDA-MB-231 and MCF-7 breast cancer cells in general was higher compared to their cytoskeletal stiffness. In contrast, the nuclear stiffness of adhesive MCF-10A human mammary epithelial cells was decreased compared to their cytoskeletal stiffness. The nuclear and cytoskeletal stiffness of MDA-MB-231 cancer cells under TSA treatment were both pronouncedly increased compared to controls, whereas the nuclear and cytoskeletal stiffness of MCF-7 cancer cells were both pronouncedly decreased compared to controls. However, the stiffness of the cytoskeleton and the nucleus of MCF-10A were not altered significantly by TSA treatment.

Thirdly, we probed whether the nuclear deformability is altered by cytoskeletal components, such as the actin cytoskeleton, and that this is based on the contractile state of the cell. Therefore, we performed inhibition assays where either the actin polymerization (Latrunculin A) or myosin activity (Blebbistatin) was impaired and measured nuclear stiffness. In fact, the nucleus of MDA-MB-231 cells was softer, when the actin polymerization was inhibited by Latrunculin A, but remained unchanged after inhibition of myosin with Blebbistatin. In addition, after combined inhibition of actin polymerization and chromatin de-condensation by latrunculin A and TSA treatment, the nucleus became significantly softer, whereas combined inhibition of myosin and chromatin de-condensation by Blebbistatin and TSA treatment did not alter nuclear stiffness. These results suggest that the stiffening of the nuclei of MDA-MB-231 cells under TSA treatment was only an apparent stiffening, probably caused by a stiffening of the cytoskeleton, which in turn compensated the somewhat softer nucleus.

## Results

### Structural and Mechanical Characterization of Two 3D Models for Cell Invasion

**FIGURE 1:**
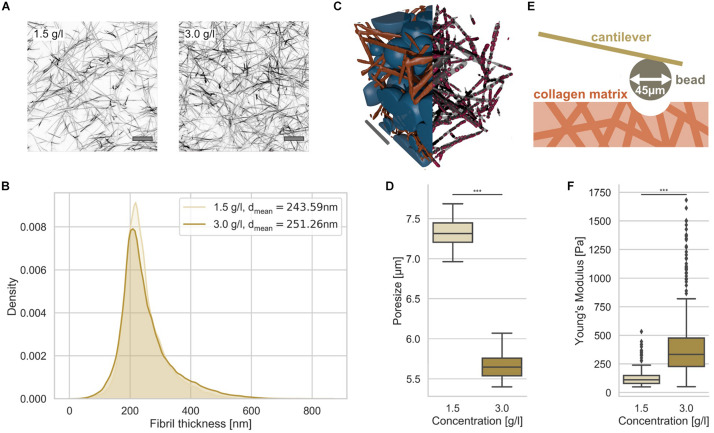
Characterization of 3D collagen matrices. **(A)** Representative image slices of TAMRA fluorescently labeled loose and dense 3D collagen networks. Scalebar is 10 μm. **(B)** Collagen fibril thickness histograms of the loose (1.5 g/l) and dense (3.0 g/l) 3D collagen matrices with mean. **(C)** Illustration of 3D matrix segmentation with collagen fibrils (orange) and detected pores (blue) (left half), fibrils in gray and measurement points in red (right half). Scale bar is 20 μm. **(D)** Pore-size values of loose and dense matrices. **(E)** Illustration of AFM-based matrix stiffness assay. The cantilever carries a bead with a diameter of 45 μm. **(F)** Matrix stiffness of loose and dense matrices determined with AFM. ***p < 0.001.

Since we seek to investigate the influence of the nuclear elasticity on cell migration in differently confined matrices, we therefore generated two types of matrices, loose and dense 3D collagen matrices, consisting of a 1:2 mixture of rat tail and bovine skin collagen type I (Mierke et al., 2018; Fischer et al., 2019; Kunschmann et al., 2019). We have chosen a collagen concentration of 1.5 g/l for the loose matrices and 3.0 g/l for the dense matrices (Figure 1A). As it is clearly visible from the representative laser scanning microscopic images, the structure is much denser in the higher concentrated matrices compared to lower concentrated matrices (Figure 1A).

In order to determine whether the collagen fiber thickness is altered due to the collagen concentration, we analyzed the fiber thickness using a slightly modified algorithm, as it has been employed similarly for the analysis of pore sizes (Fischer et al., 2019) (Figure 1B). The collagen fiber thickness (for illustration see Figure 1C right half) distribution exhibited no large difference between the two matrices (Figure 1B). In fact, the collagen fiber thickness of the two collagen matrix types were 244 ± 67 nm (*n* = 29201 collagen fiber measurement points) and 251 ± 85 nm (*n* = 59715 collagen fiber measurement points) for loose and dense matrices, respectively (Figure 1B) indicating that the collagen fibril diameter is not significantly dependent on the collagen monomer concentration. In order to determine the pore size, we fitted spheres into the 3D collagen fiber scaffold (Figure 1C, left half). The pore size of the two 3D collagen matrices was characterized using the residual pore size detection approach (Figure 1D) (Fischer et al., 2019). The loose matrix possesses a significantly larger pore size of 7.3 ± 0.2 μm (*n* = 10 collagen matrices) compared to the dense matrix with a pore size of 5.7 ± 0.2 μm (*n* = 10 collagen matrices) (Figure 1D). Both 3D migration model matrices represent restrictive cell invasion systems, since the mesh sizes of the two matrices are much smaller than the cell’s nuclear diameter. In order to validate these results, we performed a different approach, in which scanning electron microscopic images of 3D collagen fiber matrices were used to determine the pore size and the fiber thickness (Supplementary Figure S1). These results were in the similar range, but decreased due the reported shrinkage of collagen fiber samples.

The matrix stiffnesses of the two collagen matrices were determined using an atomic force microscope (AFM) with a cantilever to which a 45 μm bead was glued (Figure 1E). The elasticity (synonymously termed the Young’s modulus) of the loose matrix with 129.20 ± 75.49 Pa (*n* = 279) was pronouncedly decreased compared to that of the dense matrix with 398.03 ± 258.41 Pa (*n* = 605) (Figure 1F). Finally, we established two 3D extracellular matrices of different confinement strength for cell invasion that differ in their pore size and structure, but not pronouncedly in their fiber thickness.

### Effect of Nuclear Stiffness on Human Breast Cancer Cell Migration in Loose and Dense 3D Collagen Networks

**FIGURE 2:**
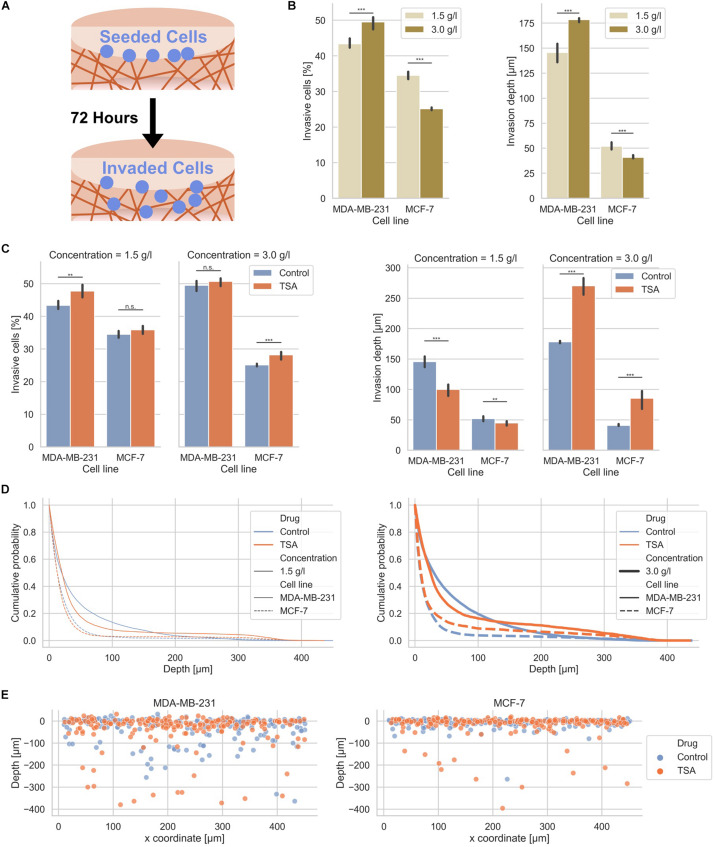
Effect of matrix confinement and TSA-treatment on the invasiveness of the two breast cancer cell types. **(A)** Illustration of the invasion assay in collagen matrix scaffolds. **(B)** Invasiveness is presented as percentage of invasive cells and invasion depth for human MDA-MB-231 breast cancer cells and human MCF-7 breast cancer cells for loose (1.5 g/l) and dense collagen matrices (3.0 g/l). Note: these results are the same as the control conditions for TSA-treatment in **(C)**. **(C)** Effect of TSA on the percentage of invasive cells and their invasion depth. **(D)** Cumulative probability as a comparative invasion histogram for MDA-MB-231 and MCF-7 on loose (left) and dense matrices (right). **(E)** Exemplary cell positions are presented in *x*–*z* direction for MDA-MB-231 (left) and MCF-7 cells (right) on dense matrices. **p < 0.01, ***p < 0.001 and n.s. not significant.

In order to investigate whether MDA-MB-231 and MCF-7 human breast cancer cells exhibit a different invasiveness into differently confined 3D extracellular matrices, such as loose and dense collagen fiber matrices, we seeded the two cell types individually on top of the two types of collagen matrices, let them adhere and invade for 3 days (Figure 2A). In fact, MDA-MB-231 breast cancer cells invaded at a higher percentage of invasive cells, such as 43.37 ± 1.11% in loose and 49.50 ± 1.39% in dense collagen gels than MCF-7 breast cancer cells, which invaded at a lower percentage of invasive cells, such as 34.52 ± 0.84% in loose and 25.14 ± 0.37% in dense collagen gels (Figure 2B). In summary, these results indicate that MDA-MB-231 cancer cells invade more numerous into dense collagen gels compared to MCF-7 cancer cells. These results were in line with our previous experiments, but due to batch to batch variations of the two collagen solutions (rat and bovine), the values differ (Mierke et al., 2008, 2011a; Mierke, 2011; Fischer et al., 2017). However, these experiments serve as control here.

Since it has been reported that the migration and invasion of cells depends on the nuclear deformability (Friedl et al., 2011; Davidson et al., 2014; McGregor et al., 2016; Thiam et al., 2016; Mierke, 2017), we determined the effect of altered nuclear mechanics on the invasion of cancer cells into two different types of 3D collagen fiber matrices. However, other studies employed for example *trans*-well assays (Rudzka et al., 2019) in contrast to the 3D collagen model system used in this study. These 3D collagen matrices constituted a more physiologically comparative extracellular matrix model system with protein compositions and topological features as well as mechanical properties comparable to a more “natural” extracellular matrix (Fischer et al., 2017), in contrast to *trans*-well assays. In detail, we analyzed the percentage of invasive cells and the invasion depths of MDA-MB-231 and MCF-7 cancer cells after 3 days in the presence of 900 ng/ml Trichostatin A (TSA), which inhibits the histone deacetylases I and II through the prevention of the removal of acetyl groups from lysine residues of histone tails (hyperacetylation) and thereby causes nuclear chromatin de-condensation (Masaeli et al., 2016; Golfier et al., 2017) (Figures 2C–E). In order to validate whether this concentration of TSA has an impact on chromatin, we analyzed the cell nuclei regarding chromatin variations with an algorithm described in Krotkov (1988) and Huang and Jing (2007) inside 3D collagen fiber matrices (invasive cells) and on top of these matrices (adherent cells) (Supplementary Figure S2). As expected, the chromatin variation was enhanced in the two cell lines after TSA treatment indicating that TSA impacts the nuclear organization. Additionally, we measured the nuclear size and chromatin variations over 17 h on a 2D substrate (Supplementary Figure S6 and Supplementary Video 1).

After TSA treatment, the percentage of invasive cells was slightly increased in loose matrices and not significantly altered in dense matrices (Figure 2C, left bar graphs). The invasiveness of MCF-7 was also only minor affected after TSA treatment (Figure 2C, left bar graphs). Hence, the invasive capacity of the two cancer cell types was slightly increased by 1.02-fold up to 1.12-fold. Although the increases in invasiveness were statistically significant, they were not considered as a substantial change. These results were not largely altered by combination of TSA with the matrix-metalloproteinase inhibitor GM6001 (Supplementary Figure S3). Moreover, GM6001 did not alter the differences between loose and dense 3D collagen fiber matrices compared to controls (Supplementary Figure S3). However, compared to controls, the usage of GM6001 greatly reduced the cells’ ability to invade into the 3D collagen matrices, as they are no longer able to degrade the matrices with MMPs (Supplementary Figure S3). Finally, the drastically reduced invasiveness of GM6001 treated cells was in turn drastically increased when combined with TSA (Supplementary Figure S3).

The invasion depth of the MDA-MB-231 cancer cells was 145.60 ± 7.80 μm in loose collagen gels and even more increased (178.40 ± 2.19 μm) in dense collagen gels and still both invasion depths were significantly higher than the invasion depths of the MCF-7 cancer cells with 52.00 ± 2.83 μm in loose collagen gels and 40.80 ± 1.79 μm in dense collagen gels (Figure 2C, right bar graphs). In loose collagen matrices, the invasion depth of both MDA-MB-231 and MCF-7 breast cancer cells was significantly reduced (Figure 2C, right bar graphs). Conversely, the invasion depth of MDA-MB-231 cells was significantly increased in dense collagen matrices to 270.40 ± 13.45 μm (1.52-fold change) and similarly the invasion depth of MCF-7 cells was significantly increased in these dense matrices to 85.60 ± 13.45 μm (2.10-fold change) (Figure 2C, right bar graphs). These results indicate that the TSA treatment seems to have noticeable effect on the invasion depth.

More precisely, the z-distribution of invaded cells can be analyzed using the cumulative probability, which shows the probability for cells to be present in a certain depth or below this depth. The cumulative probability displays the cell distribution for both cell types (treated or non-treated with TSA) in loose or dense collagen matrices (Figure 2D). Clearly, the effect of TSA on cell invasion can be seen, since more cells were present in much deeper (higher) depths inside the matrices in loose collagen matrices (Figure 2D). However, less cells were present in lower depths. Similarly, the invasion promoting effect of TSA was visible in dense 3D extracellular matrices for both breast cancer cell types (Figure 2D). In addition, the position of single cells in loose and dense collagen matrices is provided as a representatively chosen image stack of MDA-MB-231 and MCF-7 cells that had invaded for 3 days (Figure 2E). It can be clearly seen that significantly more cells of both cell types invaded in the dense collagen fiber matrices indicating that the TSA-treatment impacts the invasiveness of cancer cells in these dense matrices.

### Impact of TSA on Structural Characteristics of MDA-MB-231 and MCF-7 Breast Cancer Cells

**FIGURE 3:**
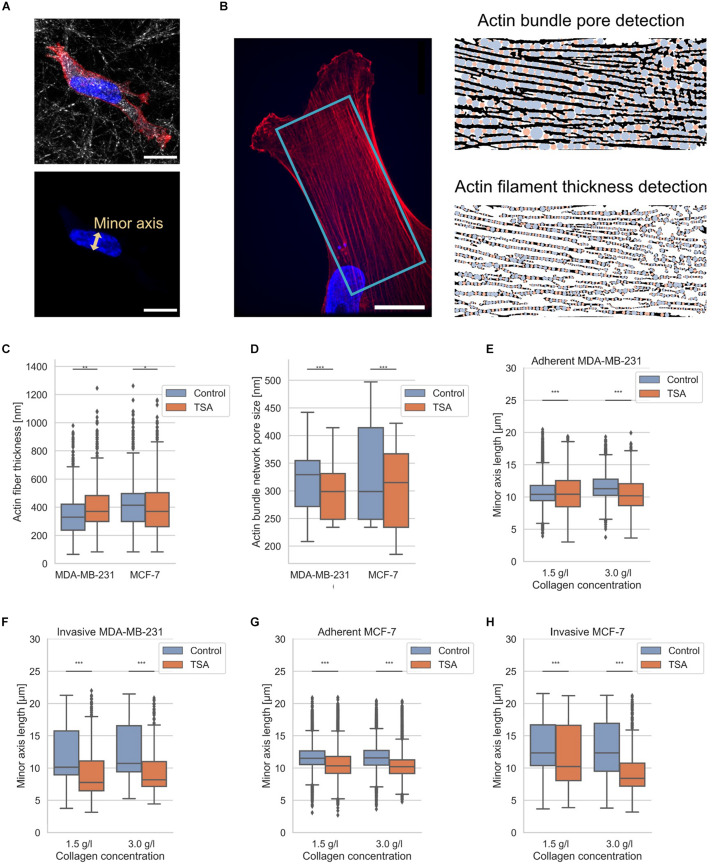
Nuclear shape and actin bundle network properties. **(A)** Exemplary MDA-MB-231 cell in 3D environment. Top confocal scanning fluorescent image of a presentative MDA-MB-231 cancer cell provided as a composite of reflection of 3D collagen fiber matrix, the actin cytoskeleton (red, Alexa Fluor 546 Phalloidin) and the nucleus (blue, Hoechst 33342). Bottom confocal scanning fluorescent image of the same representative MDA-MB-231 cancer cell for the illustration of nuclear shape parameter, the minor axis. **(B)** Illustration of determination of actin fiber bundle properties of an exemplary MDA-MB-231 cell. (Left image) Exemplary confocal laser scanning image of the actin network (red) and nucleus (blue). The analyzed image area is depicted in light blue. (Right image, top) Illustration of actin bundle network pore-size detection and (right image, bottom) fiber thickness with pores in blue and residual analysis step in orange. **(C)** Effect of TSA on actin bundle thickness. **(D)** Effect of TSA on the pore-size of the actin bundle network. **(E)** Effect of TSA on nuclear shapes of MDA-MB-231 cells that were adherent to the surface of collagen matrices and **(F)** for deeply invaded MDA-MB-231 cells. **(G)** Effect of TSA on nuclear shapes of MCF-7 cells adherent to the surface of collagen matrices and **(H)** for deeply invaded MCF-7 cells. **p < 0.01 and ***p < 0.001.

It has been reported that amounts of structural proteins are altered between MDA-MB-231 and MCF-7 breast cancer cells, such as actin (Yamaguchi and Condeelis, 2007; Mierke et al., 2011a, b; Liu et al., 2016; Tang and Gerlach, 2017). Hence, we also hypothesized that the different invasiveness of the two breast cancer cell types depends on differences in their cytoskeleton, such as structure, and on differences of cellular compartments, such as the nucleus, since it has been hypothesized that the shape of the nucleus impacts the migration of cells (Friedl et al., 2011; McGregor et al., 2016; Calero-Cuenca et al., 2018). However, whether there exist structural changes in the pore sizes of actin bundles inside cancer cells and the precise fiber thickness has not been analyzed. Since the currently available analysis programs are user-dependent, such as the manual selection of structural features with ImageJ, we presented here an automated detection program (Figure 3). Therefore, we applied our recently established technique to determine 3D pore sizes of collagen networks (Fischer et al., 2019) to now determine the 2D pore size of quasi-static actin networks (Figure 3B right top and 3D) and the thickness of the actin fibers (Figure 3B right bottom) in MDA-MB-231 and MCF-7 cells (Figure 3C).

The actin fiber thickness of MDA-MB-231 cancer cells was 351 ± 138 nm (*n* = 8061 fiber measurement points) and the actin fiber thickness of MCF-7 was 405 ± 155 nm (*n* = 13399 fiber measurement points). In the presence of TSA, the actin fiber thickness of MDA-MB-231 cancer cells increased to 405 ± 150 nm (*n* = 4500 fiber measurement points), whereas the actin fiber thickness of MCF-7 cancer cells was not significantly altered (405 ± 165 nm, *n* = 8566 fiber measurement points) (Figure 3C). These results indicate that the actin fiber thickness of MDA-MB-231 cancer cells is significantly increased due to the TSA treatment, whereas the actin fiber thickness of non-malignant MCF-7 cells is significantly decreased after TSA treatment (Figure 3C).

The actin bundle pore size was 314 ± 71 nm for MDA-MB-231 cancer cells (*n* = 900 pores) and 334 ± 88 nm for MCF-7 cancer cells (*n* = 1300 pores). After TSA treatment, the actin bundle pore sizes decreased to 299 ± 52 nm (*n* = 900 pores) for MDA-MB-231 cancer cells and 305 ± 80 nm (*n* = 1200 pores) for MCF-7 cancer cells (Figure 3D). Hence, the actin bundle network pore size was significantly decreased in both MDA-MB-231 and in MCF-7 cancer cells after TSA treatment (Figure 3D).

Moreover, since the nucleus represents an obstacle for cell invasion in 3D extracellular matrices, such as in collagen matrices, we also determined the length of the nuclear minor axis of adhesive and invasive MDA-MB-231 breast cancer cells (Figures 3E,F) and adhesive and invasive MCF-7 breast cancer cells (Figures 3G,H). We considered the minor axis of the cell nucleus as a measure of the nuclear deformation. In detail, when the minor axis length of the nucleus increased, the nucleus becomes a major obstacle for cell migration and causes a migration stalled phenotype in a confined 3D microenvironment. In line with these results, the aspect ratio of the invasive cell nuclei among the MDA-MB-231 is increased, whereas their nuclear volume is decreased indicating that the nucleus is deformed (Supplementary Figure S4). The nuclear volume decrease contrasts with previous studies (Mazumder et al., 2008; Chan et al., 2017). However, we were able to observe a nuclear volume increase on 2D substrates (Supplementary Figure S6), indicating that the cell nuclei were compressed inside our 3D collagen matrices representing narrow confinements. Furthermore, there was no linear correlation between nuclear volume and invasion depth under TSA treatment, as shown in Supplementary Figure S7, indicating that nuclear volume was not solely responsible for an increase in invasion depth under TSA treatment. As expected, the MDA-MB-231 cells possessed nuclei with a smaller minor axis length than MCF-7 cells regardless of whether the cells adhered to or penetrated the collagen matrix surface and independent of the type of the matrix (Figures 3E–H).

In detail, the nuclear minor axis length of adhesive MDA-MB-231 cells was significantly increased on loose matrices (from 10.74 ± 1.95 μm, *n* = 18405 nuclei to 10.53 ± 2.74, *n* = 6650 nuclei), but significantly decreased on dense matrices after TSA-treatment (from 11.60 ± 1.85 μm, *n* = 19990 nuclei to 10.45 ± 2.39 μm, *n* = 3533 nuclei) (Figure 3E). In contrast, the minor axis length of deeply invaded MDA-MB-231 cells (only the last 10% of the deepest invaded cells) was significantly decreased after TSA treatment in loose (from 10.61 ± 3.80 μm, *n* = 1569 nuclei to 9.14 ± 3.79, *n* = 627 nuclei) and in dense matrices (from 12.56 ± 3.94 μm, *n* = 1849 nuclei to 9.56 ± 3.66, *n* = 333 nuclei) (Figure 3E).

The TSA-treatment of adhesive MCF-7 cells decreased significantly the nuclear minor axis length on both loose (from 11.68 ± 1.95 μm, *n* = 18533 nuclei to 10.72 ± 2.47, *n* = 15483 nuclei) and dense collagen matrices (from 11.66 ± 1.94 μm, *n* = 21867 nuclei to 11.84 ± 2.05 μm, *n* = 12275 nuclei) (Figure 3G). A similar effect was observed by invaded MCF-7 that had been treated with TSA, since the nuclear minor axis length was decreased in invaded MCF-7 cells on both loose (from 13.11 ± 4.03 μm, *n* = 1297 nuclei to 11.84 ± 4.58, *n* = 1132 nuclei) and dense collagen matrices (from 13.11 ± 4.39 μm, *n* = 1522 nuclei to 9.55 ± 3.51 μm, *n* = 845 nuclei) (Figure 3H). In summary, the minor axis length of invaded cells decreased significantly, when these cells invaded in loose matrices and even more pronouncedly when they invaded in dense matrices. In general, it can be stated that TSA treatment mostly decreased the minor axis length of the cell nuclei of both cell types, independently of the matrix type, with the only exception of adhesive MDA-MB-231 cancer cells on loose matrices.

### Cytoskeletal and Nuclear Stiffness of Adherent Human Breast Cancer Cells and Healthy Mammary Epithelial Cells

If the “universal” hypothesis that individual cancer cells are generally softer than non-malignant cells is true (Guck et al., 2005; Cross et al., 2007; Remmerbach et al., 2009; Fritsch et al., 2010; Runge et al., 2014; Lekka, 2016; Meinhövel et al., 2018), MDA-MB-231 cancer cells should be softer than MCF-7 cancer cells, since the MDA-MB-231 cancer cells were more invasive compared to MCF-7 cells. But is the softness of every malignant cancer cell type independent of its biochemical or genetic phenotype fundamentally dependent on different cytoskeletal mechanics or on different nuclear mechanics or on both?

**FIGURE 4:**
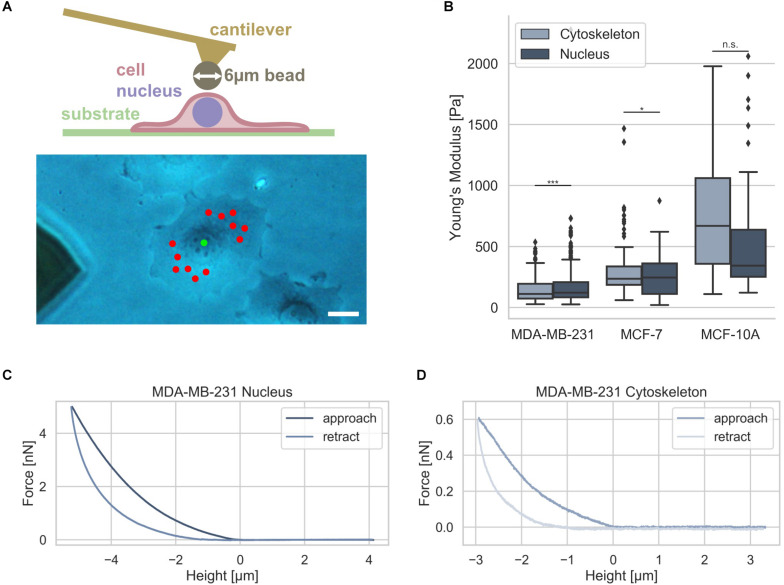
Cell stiffness measurement of MDA-MB-231 and MCF-7 breast cancer cells using AFM. **(A)** Illustration of AFM measurement technique: an individual adherent cancer cell is measured with a 6 μm bead carrying cantilever. Exemplary measurement points for the cytoskeleton and the nucleus are indicated in a phase contrast image with the cytoskeletal measurement points depicted in red and nucleus measurement point in green. Scalebar is 10 μm. **(B)** Stiffness measurements of the cytoskeleton (light gray) and the nucleus (dark gray) of MDA-MB-231 and MCF-7 breast cancer cells as well as control mammary epithelial MCF-10A cells. Exemplary force-distance curves of MDA-MB-231 cells of the nucleus **(C)** and cytoskeleton **(D)** are presented. *p < 0.05, ***p < 0.001, and n.s., not significant.

Since it has been hypothesized that the nuclear stiffness is higher than the cytoskeletal stiffness in living cells, we analyzed whether there exists a difference between these two compartments in two human breast cancer cell types, such as MDA-MB-231 and MCF-7, and mammary MCF-10A epithelial control cells. Moreover, it has been hypothesized that nuclear mechanics of diseased cells, such as cancer cells, is altered compared to normal healthy cells in a simplified manner (Friedl et al., 2011; Tariq et al., 2017). The nuclear and cytoskeletal stiffness of adhesive breast cancer cell types were determined as a reference using atomic force microscopy (AFM). The cantilever of the AFM carried a bead with a diameter of 6 μm that had been glued to it (Figure 4A, upper image). A standard measurement protocol for the cytoskeleton probing random perinuclear points with 0.5 nN was used (Fischer et al., 2017) (Figure 4A, lower image, red dots). However, to determine reliably of the nuclear stiffness, a higher maximum indentation force of 5 nN had to be applied to the cells at a single point centered above the cell nucleus (Figure 4A, lower image, green dot) (Krause et al., 2013). In the next step the force approach and retraction curves of the cell nucleus and cytoskeleton were determined and representative curves were provided for MDA-MB-231, MCF-7, and MCF-10A cells (Figures 4C,D). The MDA-MB-231 breast cancer cells possessed a significantly softer cytoskeleton with a Young’s modulus of 103.42 ± 89.45 Pa (*n* = 68 cells) compared to the Young’s modulus of the nucleus of 157.70 ± 78.55 Pa (*n* = 69 cells) (Figure 4B). However, the MCF-7 cells showed only a minimally significant difference of the cytoskeletal Young’s modulus of 394.89 ± 295.97 Pa (*n* = 34 cells) and the nuclear Young’s modulus of 399.01 ± 117.16 Pa (*n* = 55 cells) indicating that these non-malignant cells exhibit a stiffer nucleus (Figure 4B).

In order to reveal whether also human mammary epithelial cell type MCF-10A behave differently to MDA-MB-231 and MCF-7 breast cancer cells, we determined their stiffness with AFM. We found that the MCF-10A cells showed no significant difference between the cytoskeletal Young’s modulus of 690.85 ± 432.85 Pa (*n* = 20 cells) and the nuclear Young’s modulus of 594.66 ± 540.46 Pa (*n* = 20 cells) due to the large variations in both groups (Figure 4B). These results demonstrate that there is only a large significant difference between the cytoskeletal and the nuclear stiffness of the MDA-MB-231 breast cancer cells.

### Nuclear Mechanics Alterations Are Caused by the Pharmacological Drug TSA and Impact 3D Migration

**FIGURE 5:**
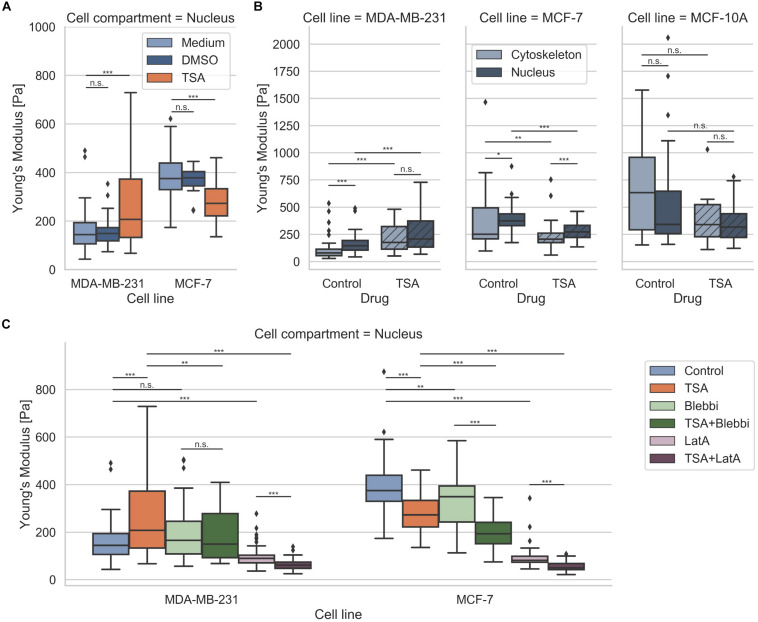
Effect of TSA on cell stiffness using AFM. **(A)** TSA buffer control measurements with malignant MDA-MB-231, non-malignant MCF-7 cancer cells. **(B)** Effect of TSA on cytoskeletal and nucleus elasticity of the human breast cancer cells MDA-MB-231 and MCF-7 human breast cancer cells an the normal human MCF-10A epithelial cells. **(C)** Interdependence of cytoskeletal mechanics and nuclear mechanics based on the acto-myosin cytoskeleton. MDA-MB-231 and MCF-7 cancer cells were treated with TSA, the myosin inhibitor Blebbistatin (Blebbi), the actin polymerization inhibitor Latrunculin A (LatA) or combined treatments of Blebbi with TSA and LatA with TSA. *p < 0.05, **p < 0.01, ***p < 0.001, and n.s., not significant.

Since it has been reported that a chromatin remodeling drug, such as TSA, impacts the nuclear volume (Chan et al., 2017), we investigated the effect of 900 ng/ml TSA on cellular stiffnesses using AFM (Figure 5). TSA treatment increased cytoskeletal and nuclear stiffness of the MDA-MB-231 cancer cells from 103.42 ± 89.45 Pa (*n* = 68) to 221.30 ± 121.64 Pa (*n* = 54) and from 157.70 ± 78.55 Pa (*n* = 69) to 262.69 ± 160.64 Pa (*n* = 91), respectively. In contrast, TSA treatment decreased the cytoskeletal and nuclear stiffness of the MCF-7 cancer cells from 394.89 ± 295.97 (*n* = 34) to 233.19 ± 128.26 Pa (*n* = 40) and from 399.01 ± 117.16 Pa (*n* = 55) to 282.14 ± 85.16 Pa (*n* = 54), respectively (Figure 5A). In line with this result, TSA decreased the cytoskeletal and nuclear stiffness of the human mammary MCF-10A epithelial cells from 690.85 ± 432.85 Pa (*n* = 20) to 433.09 ± 333.27 Pa (*n* = 6) and from 594.66 ± 540.46 Pa (*n* = 20) to 353.82 ± 178.93 Pa (*n* = 28), respectively (Figure 5A). There were no significant differences in the nuclear stiffness of the two cancer cell types, MDA-MB-231 and MCF-7, when cultured in DMSO containing medium (buffer control) indicating that the effect after TSA-treatment is solely drug specific (Figure 5B). These results indicate that the MDA-MB-231 cancer cells behaved differently possibly due to the de-condensation of chromatin, alterations in gene expression (which seem to be rather unlikely on such small timescales) or an effect of TSA on the cytoskeletal structure and dynamics.

In general, we found that the stiffness of the nuclei of cancer cells were pronouncedly higher compared to their cytoskeleton, which seems to be independent of the cell line. However, when the cells were treated with TSA, the two cancer cell lines exhibited a different behavior: the stiffness of the nucleus of MDA-MB-231 cells was increased, whereas the stiffness of the nucleus of MCF-7 cells was decreased. The treatment of human mammary epithelial cells with TSA, led to pronouncedly decreased nuclear stiffness, hence their behavior is similar to the human MCF-7 breast cancer cells. Similar results were obtained, when the MDA-MB-231 and MCF-7 cells were treated with the GM6001 or a combination of GM6001 and TSA (Supplementary Figure S5) indicating that TSA has the same effect in the absence of matrix-metalloproteinases than in the presence.

### Are Nucleus Mechanics Compensated by Cytoskeletal Actin Stiffening in Human MDA-MB-231 Breast Cancer Cells?

The nucleus is embedded within the cell’s cytoskeleton and hence its mechanical properties are regulated by the cytoskeletal mechanics (Mierke, 2017; Uhler and Shivashankar, 2018; Wang et al., 2018) and the matrix environmental mechanics (Lammerding, 2011; Martins et al., 2012; Haase et al., 2016). We analyzed whether the stiffening of the nucleus of MDA-MB-231 cells under TSA treatment is dependent on cytoskeletal mechanics. More precisely, we hypothesized that the nuclear stiffening of MDA-MB-231 breast cancer cells may be evoked by a stiffening of the actin cytoskeleton, which may be based on a cytoplasmic compensatory mechanism for the TSA-induced softening of the nuclei. To investigate whether the acto-myosin cytoskeleton causes the stiffening of the nuclei after TSA treatment, we modulated the acto-myosin cytoskeleton by inhibition of myosin. Therefore, we reduced the myosin II motor protein affinity to the filamentous actin network by treating cells with 25 μM Blebbistatin (Figure 5C) as it has been reported (Shutova et al., 2012; Streppa et al., 2018). In fact, a slight but not significant stiffening of the nucleus of MDA-MB-231 cancer cells can be observed (from 157.70 ± 78.55 Pa with *n* = 69 cells to 191.52 ± 107.52 Pa with *n* = 59 cells) after Blebbistatin treatment (Figure 5C). Moreover, the combined treatment of Blebbistatin and TSA showed no significant further increase in stiffness 190.64 ± 104.28 Pa (*n* = 59) (Figure 5C). In order to explore the effect of the actin filaments on nuclear stiffness, we treated MDA-MB-231 breast cancer cells with 0.2 μM Latrunculin A, which effectively eliminates actin filaments (Fischer et al., 2017; Kunschmann et al., 2019). In fact, we observed a significant softening of the nucleus to 95.02 ± 40.89 Pa (*n* = 72) (0.60-fold change; Figure 5C). We hypothesized that this residual nuclear stiffness is now largely determined by the cell nucleus itself, since the effect of the actin cytoskeleton is abolished by the Latrunculin A treatment. When MDA-MB-231 cancer cells undergo a combined treatment of Latrunculin A and TSA, the predicted nuclear softening can be observed, since the nuclei soften to 64.49 ± 23.78 Pa (*n* = 54) (0.41-fold change) (Figure 5C). Finally, these results indicate that the acto-myosin cytoskeleton seems to impact nuclear mechanics and may even convert the apparent stiffening effect of TSA on MDA-MB-231 cell nuclei to a softening effect by combined treatment with the actin polymerization inhibitor Latrunculin A.

In contrast, the TSA-treatment of MCF-7 cells did not induce a stiffening of the nucleus, instead a softening of the nucleus occurred. The addition of Blebbistatin or Latrunculin A increased significantly the nuclear softness of MCF-7 cells (Figure 5C). The addition of TSA to Blebbistatin or Latrunculin A-treated cells further increased nuclear deformability (Figure 5C). In contrast to MDA-MB-231 cancer cells, the addition of TSA caused a softening of the nucleus that is even more pronounced in the presence of myosin or actin polymerization inhibitors. These cell-type specific effects of the TSA-treatment revealed that there are differences between cancer cell types toward the effect of drug treatment, such as TSA. Moreover, these differences may correlate with the invasive capacity or cytoskeletal rearrangements of the cell types.

## Discussion

The cell migration and invasion depend on the nuclear and cytoskeletal mechanics, which are interdependent and thereby possibly impact each other’s mechanical properties. It has been demonstrated that cytoskeletal mechanics can alter the nuclear mechanics (Mierke, 2017; Uhler and Shivashankar, 2018; Wang et al., 2018). In accordance with these findings, the matrix mechanics of the cellular microenvironment can additionally alter the cytoskeletal mechanics, which will then subsequently change the nuclear mechanics (Lammerding, 2011; Martins et al., 2012; Haase et al., 2016; Rabineau et al., 2018). As the nucleus is the largest and stiffest organelle in the cell, it is an important limiting factor for the migration of cancer cells through dense connective tissue. Under none matrix-degrading conditions (presence of the MMP inhibitor GM6001), the size of the nucleus seems to be critical for the migration and invasion of cells, since the physical hindrances for motility in 3D, such as a dense 3D collagen fiber network can no longer be degraded. Hence, under inhibition of matrix-metalloproteinases, the nuclear size may even represent a larger steric obstacle for cell migration and invasion, when the cellular environment is more confined, such as in a dense matrix compared to a loose matrix. In specific detail, when the cell surrounding collagen fiber matrix can no longer be degraded, either the cell nucleus or the surrounding matrix needs to undergo physical deformation during cell migration. A major constituent of the nucleus is chromatin. Similar to any polymer, chromatin stiffens upon an increase in concentration through condensation (Pajerowski et al., 2007; Irianto et al., 2016). Hence, the chromatin state, such as condensed or de-condensed, seems to be a key factor for the migration and invasion of cancer cells through matrix confinements. In fact, we were able to soften the nuclei after TSA treatment of MCF-7 and in principle also of MDA-MB-231 cells. However, this effect of the nuclear softening may not be visible in MDA-MB-231 cells, as it seems to be possibly compensated by the cytoskeletal stiffness, which has been increased after TSA treatment. Moreover, other not yet identified mechanisms based on a direct TSA effect on the actin cytoskeleton or rather unlikely (due to small time scales for the stimulation) on TSA-based altered gene expression may have impaired a nuclear softening in MDA-MB-231 cells. In addition, we observed a decrease in migration speed of both MDA-MB-231 and MCF-7 cells on 2D substrates devoid of any steric hindrances in the presence of TSA (Supplementary Figure S8). Thus, the nuclear mechanics represent no obstacle for the migration of cells in 2D. In contrast to the 2D situation, a pronouncedly increased invasion depth in dense 3D collagen matrices (representing steric hindrances) after TSA treatment can be observed in the presence (no degradation of the matrix) and absence of MMPs (with matrix degradation). These findings further justify the hypothesis that nuclear mechanics seems to be a key regulator of cell invasion.

Previous studies focused on the mechanical interaction of cells and their microenvironment (Staunton et al., 2016) or studied nuclear mechanical properties *ex situ* (Doss et al., 2015). Both studies investigated similar aspects of cellular and nuclear properties and cell invasion. However, in our study, a drastically different collagen model was utilized, and novel approaches and methods were employed to examine nuclear stiffness and cancer cell invasion. The effect of TSA on nuclear size of isolated cell nuclei *ex situ* have been studied previously (Mazumder et al., 2008; Chan et al., 2017). In contrast to these studies, we found a nuclear volume decrease for cells inside the narrow confinements of our 3D collagen model system when TSA was applied, which seems to have limited the nuclear expansion under TSA treatment and instead lead to a compression, which is an interesting discrepancy that needs to be studied in the future.

For 3D migration model systems, collagen type I matrices have been established, since they mirror the mechanics of natural extracellular matrices that we improved by using a mixture of two volumes bovine skin and one volumes rat tail collagen to generate matrices that are on the one hand more similar to the extracellular matrix of tissues (Paszek et al., 2005) and on the other hand less variable due to batch variations (Kreger et al., 2010). The mixture also shows stiffnesses in the range of connective tissue (Lang et al., 2015), which renders it more suitable to mimic cancer cell migration *in vivo*. Irreversible, plastic deformations as observed in previous studies by applying forces in the range of several μN (Wolf and Friedl, 2011; Mohammadi et al., 2015) seem to be negligible here, due to the drastically different mechanical properties of our collagen matrices and those collagen matrices used in those studies. Moreover, cells generate forces in the range of nN (Thievessen et al., 2015), which are a magnitude smaller than the μN forces applied toward the collagen matrices in these studies. However, these cellular forces may still be high enough to possibly plastically deform the matrices, which may have a little effect on the cellular motility in 3D, as it has been reported (Koch et al., 2012).

The difference between loose and dense 3D collagen fiber matrices is mainly based on pore size and matrix stiffness and not on collagen fiber thickness, where the dense collagen fiber matrices possess smaller pores and exhibit higher stiffness, but display a similar collagen fiber thickness as loose collagen matrices. The fiber thickness analysis is considered to be overestimated, although post-deconvolution and multiple post-processing steps allowed an analysis with length scales below the optical diffraction limit. The proposed analysis algorithm determines fiber diameters in a pixel-wise manner with a theoretical resolution limit similar to the voxel size of the images. We have verified these values using an electron scanning microscope leading to a comparable fiber thickness. However, this analysis must still be considered as an estimation similar as it has been reported in Franke et al. (2014). These findings correspond to the results on pure rat 3D collagen fiber matrices of different collagen concentrations, where the collagen fibril diameter is not significantly dependent on the collagen monomer concentration (Sapudom et al., 2015). Hence, these two matrices represent appropriate model systems for cancer cell invasion that confine cell invasion on the nuclear level due to the small pore sizes. Here, the collagen fibril diameter of our 2:1 mixture of bovine to rat collagen type I matrices is smaller than that of pure rat collagen type I (Sapudom et al., 2015).

As expected, firstly, the loose 3D collagen matrices seem to be less restrictive for cell migration and invasion of cancer cells, such as MDA-MB-231 and MCF-7 human breast cancer cells, since the nucleus is less confined and hence will not require large nuclear deformation, which we observed. The invasiveness of these two human breast cancer cell lines have been determined previously (Frixen et al., 1991; Guck et al., 2005; Mierke et al., 2008). Secondly, the dense 3D collagen matrices seem to be more restrictive for cancer cell migration and invasion, since the cell nucleus will require increased nuclear adaptation. These results were in line with our previous experiments and serve as control here (Mierke et al., 2008, 2011a,b; Fischer et al., 2017). However, due to a change in collagen I monomer batch, the properties of the reconstituted collagen matrices changed slightly and hence the values for invasiveness and invasion depth are not directly comparable.

When investigating the nuclear stiffness of TSA-treated cells, we found that the nuclear stiffness of MDA-MB-231 breast cancer cells was apparently increased. The cytoskeleton of MDA-MB-231 cells drastically stiffened under TSA treatment, which led to increased stiffness values at the nucleus measurement points using AFM, although the nucleus have been theoretically softened due to TSA addition. Hence, we verified the actual softening of the nucleus by further investigating TSA treatment using pharmacological drugs. However, in line with the MDA-MB-231 cell results, we showed that the nuclei of TSA-treated MCF-7 cells were softened and the invasion depths of the cells were even significantly increased in dense 3D collagen matrices. Their penetration depths into loose and hence less confined 3D collagen matrices were reduced after induction of chromatin de-condensation.

Since the nucleus is mechanically connected to the surrounding microenvironment by linker of nucleoskeleton and cytoskeleton complex (LINC) proteins (Crisp et al., 2006) that are linked to the inner and outer nuclear membranes, the nucleus is coupled to the contractile cytoskeleton, which can alter the extracellular matrix or surrounding cellular environment through focal adhesion remodeling (Martins et al., 2012; Belaadi et al., 2016; Heo et al., 2016a, b). Subsequently, the nuclear envelope is physically connected to the contractile cytoskeleton. Hence, we hypothesize that for both cancer cell types a nuclear softening may occur after chromatin de-condensation, but it seems to be counterbalanced by the individual cytoskeleton for distinct cell types, such as those with pronounced actin fibers that create a strong acto-myosin network deforming the matrices (Fischer et al., 2017).

Moreover, it has been reported that alterations in cytoskeletal mechanics, such as perinuclear cytoskeletal mechanics are crucial for cell functions, such as adaptation to external confinement mechanics changes (Miroshnikova et al., 2017). In addition, chromatin mechanics can be adapted to mechanical cues and thereby influence cellular functions to preserve the DNA from damage.

Cells are continuously subject to dynamic changes in their external microenvironmental mechanics, and therefore, a biochemical rearrangement of cell-matrix coupling and a physical restructuring of multiple cellular compartments, such as the nucleus, is necessary. Since the nucleus contains nearly the entire DNA of the cells, it is the largest and properly the stiffest structure inside the cells (Dundr and Misteli, 2001; Dahl et al., 2004; Misteli, 2007; Booth-Gauthier et al., 2012). Thus, the nucleus represents a major obstacle for cell invasion into confined matrices. In detail, chromatin remodeling is facilitated by several post-translational modifications, such as methylation and acetylation, that enables shifts between densely packed chromatin (heterochromatin) and loosely packed (euchromatin) representing an epigenetic regulation (Laugesen and Helin, 2014; Tessarz and Kouzarides, 2014). Hence the nuclear mechanics, such as stiffness, seems to be regulated by the state of chromatin, such as condensed and decondensed altering cellular functions, such as migration and invasion. Moreover, the nuclear mechanics may be altered on longer timescales, such as a few hours, by altered gene expression. Nuclear softening is also induced during the migration of cells through confinements (Cao et al., 2016; Denais et al., 2016; Thiam et al., 2016).

As we did not observe a nuclear stiffening in MDA-MB-231 cells treated with both TSA and Latrunculin A, we hypothesize that the acto-myosin cytoskeleton, such as perinuclear actin, may impair nuclear softening and even induce nuclear stiffness after TSA treatment. Actin-dependent mechanical adaptation and stress dissipation mechanisms, such as deformation, in the cytoskeleton and the nucleoskeleton are responsible for providing cell invasion and protection of genetic material representing essential hallmarks of cancer (Miroshnikova et al., 2017). Hence, nuclear rupture has been detected frequently during migration and invasion through confinements (Harada et al., 2014; Denais et al., 2016; Raab et al., 2016; Thiam et al., 2016). A more elastic nucleus with perinuclear F-actin fibers and reduced lamin A/C at the lamina has been reported to protect cells from nuclear rupture (Cao et al., 2016; Denais et al., 2016; Thiam et al., 2016). Here, we have shown that the cytoskeletal mechanics of MDA-MB-231 cancer cells seems to counterbalance a softening of the nucleus by addition of TSA, which is probably based on the actin polymerization-based cytoskeleton. Moreover, when the nucleus of MDA-MB-231 is treated with TSA, the chromatin is decondensed switching the nucleus to a more viscous material. However, when the polymerization of the actin cytoskeleton is inhibited additionally, both treatments soften the nucleus more than their separated treatments. The results were obtained with exactly the same analysis parameters and residual RMS threshold for all recorded force-distance curves, regardless of the cell line and drug treatment. In contrast to MCF-7 cancer cells, in which the actin stress fibers are less pronounced, the TSA-induced softening of the nucleus cannot be counterbalanced or rescued by the actin filaments. As a result, MCF-7 cancer cells possess a softer nucleus after TSA treatment.

A differential viscosity of heterochromatin and euchromatin has been found by using the histone deacetylase inhibitor trichostatin A, which decondenses chromatin (Spagnol and Dahl, 2016). More precisely, the nuclear interior is more viscous and deformable after decondensation of the dense heterochromatin (Pajerowski et al., 2007; Chalut et al., 2012; Spagnol and Dahl, 2016). Besides TSA treatment, the mechanical strain can facilitate the decondensation of heterochromatin (Le et al., 2016) indicating a dynamic coupling of chromatin density (packing) and nuclear mechanics (Pagliara et al., 2014). In rather undifferentiated cells, such as cancer cells, mechanical perturbation induces nuclear deformation together with cellular deformation (Heo et al., 2016a). Chromatin thus regulates cell and nuclear mechanics by acting as a viscoelastic element of the nucleus that dynamically changes its condensation state due to altered mechanics (Miroshnikova et al., 2017) and enables guided cell migration based on dynamically fluctuating (tugging) tractions (Plotnikov and Waterman, 2013). In addition, the decondensation may induce the expression of genes involved in the regulation of nuclear or cytoskeletal mechanical properties.

In turn, environmental mechanics, such as matrix mechanics (Ingber, 2003; Huang and Ingber, 2005; Levental et al., 2009; Laklai et al., 2016; Miroshnikova et al., 2017) and the chromatin state (Swift et al., 2013; Denais et al., 2016; Heo et al., 2016a, b; Le et al., 2016) are dynamically linked and thereby alter cellular functions (Barriga et al., 2018). However, this connection seems to be impaired in diseases, such as cancer. Since TSA treatment altered the chromatin state, we see changes in the mechanical properties. In addition, as we have seen cytoskeletal alterations of TSA treatment, such as a thickening of the actin bundles in MDA-MB-231 cells, hence it seems to be possible that the TSA-induced softening of the nucleus is stabilized by the surrounding actin cytoskeleton and consequently, the nucleus appears even stiffer. In line with the thickening of the actin bundles, the actin bundle network pore size decreases pronouncedly. In contrast, we did not observe a thickening of actin bundles in MCF-7 cells, which seems to be an explanation why these nuclei were softer after TSA treatment. Hence, the softening of MCF-7 nuclei appears not to be counter-balanced by the acto-myosin cytoskeleton.

Finally, although this study reveals distinct perinuclear actin cytoskeletal systems of the two breast cancer cell types, both cell types require to disassemble the stiff nuclear lamina to circumvent steric obstacles of their matrix environment during the migration and invasion of the cell. Hence, the nucleus can be seen as a cell compartment limiting cell movement in 3D extracellular matrix confinements even in the presence of matrix-degrading enzymes.

Key findings (impact on science):

1. Young’s moduli of the nucleus and the cytoskeleton of MDA-MB-231 and weakly invasive MCF-7 cancer cells are altered.
2. Nuclear deformability affects 3D migration of breast cancer cells.
3. Chromatin decondensation increases invasion depths of breast cancer cells in dense 3D matrices.
4. Nuclear chromatin decondensation leads to increased cytoskeletal stiffness in MDA-MB-231 cancer cells, resulting in an apparent stiffening of the nucleus, but not in MCF-7 cancer cells.
5. Acto-myosin dependent cytoskeletal stiffness seems to regulate nuclear stiffness.

## Materials and Methods

### Cells and Cell Culture

Human breast cancer cell lines, such as MDA-MB-231 and MCF-7, and the human mammary epithelial cell line MCF-10A were purchased from ATCC-LGC-Promochem (Wesel, Germany). These cancer cell lines were cultured in 4.5 g/l DMEM with added 10% Fetal Calf Serum and 1% penicillin-streptomycin (Biochrom, Berlin, Germany) in an incubator at 37°C, 5% CO_2_ and 95% humidity. Instead, the mammary epithelial cell line MCF-10A was cultured in a 1:1 mixture of DMEM (4.5 g/l glucose, L-glutamine) and Ham’s F12 medium with added 5% Horse Serum, 1 g/l Cholera toxin stock solution, 10 mg/ml Insulin (Sigma-Aldrich, I9278), 1 g/l Hydrocortison stock solution, 100 μg/ml epidermal growth factor (EGF) and 1% penicillin/streptomycin 100× (P/S) under the same conditions as mentioned above. For all experiments, cells with passage numbers of 5–25 at about 80% confluency were harvested.

### 3D Collagen Matrices

These extracellular matrix models are comprised of a mixture of rat tail (4 mg/ml rat collagen type I, Serva, Heidelberg, Germany) and bovine skin (4 mg/ml bovine collagen type I, Biochrom, Berlin, Germany) collagen monomers at a mass fraction of 1:2, respectively (Paszek et al., 2005; Lang et al., 2015). The monomer solution was polymerized using a 1 M phosphate buffered solution containing sodium dihydrogen phosphate (Sigma-Aldrich, Cat. No. 71507), disodium hydrogen phosphate (Sigma-Aldrich, Cat. No. 71636) and ultrapure water, mixed to a pH value of 7.4 and ionic strength of 0.7 and final phosphate concentration of 200 mM. All components were kept at 0°C during mixing (Fischer et al., 2017, 2019; Kunschmann et al., 2017, 2019). Finally, the cooled solution was added to a 6-well plate for invasion assays, or μ-Plates for analyses of the pore sizes and fibril thicknesses. The extraction method of commercially available collagen I monomers influences the structure of the collagen networks and their assembly kinetics (Wolf et al., 2009; Kreger et al., 2010; Parenteau-Bareil et al., 2010). The rat and bovine mixture of a 1:2 monomer mass ratio has been shown to provide more physiological elastic properties than pure rat tail collagen matrices (Paszek et al., 2005; Antoine et al., 2014; Lang et al., 2015).

### Analysis of the Pore Size and Fibril Thickness in 3D Matrices

Collagen matrices are prepared with the same mixture of the two collagen monomer solutions as described above. More precisely, 500 μl monomer solution is filled in each well of an ibidi 24-well μ-Plate und polymerized in an incubator at 37°C, 95% humidity for 2 h. These collagen matrices were fluorescently stained using 20 μg/ml 5(6)-Carboxytetramethylrhodamine *N*-succinimidyl ester (TAMRA-SE) (Sigma-Aldrich, Cat. No.: 21955) for 24 h. Subsequently, these matrices were washed 3 times using 1 ml PBS and stored in 1 ml PBS at 4°C. The stained gels were imaged using a confocal laser scanning microscope (Leica TCS SP8, Mannheim, Germany) with a 40× NA/1.10 water immersion objective. 3D image cubes with edge length of 150 μm were recorded and analyzed using a custom-built python program, as described in Fischer et al. (2019). The pore size is defined as the median pore-diameter of a particular, analyzed sample. The collagen fibril diameter was determined using a modification of the algorithm presented in Fischer et al. (2019). In more detail, firstly, we recorded 3D image stacks with an edge length of 25 μm using a 63× water immersion objective, resulting in high resolution images of collagen fibrils. Secondly, we calculated the precise binary as described in Fischer et al. (2019) to get a segmentation of collagen fibrils and fluid phase. Subsequently, we applied the same algorithm as in Fischer et al. (2019), but on the collagen phase, not the fluid phase. Hence, we have an estimation of the fibril thickness, instead of the size of the pores. Contrary to Fischer et al. (2019), a single analysis step was sufficient. No residual analysis steps were carried out. As a result, we obtain several measurement points that were distributed along each fibril representing the 3D fibril diameter at each individual measurement point (Figure 1C, right half).

### Analysis of the Matrix Elasticity

To determine collagen matrix mechanical properties, we used an AFM as published previously (Fischer et al., 2017). In detail, a 45 μm polystyrene bead was glued to the lower side front of a tip-less cantilever. In the next step, this large bead was indented into the surface of a polymerized collagen matrix with a maximum indentation force of 5 nN (Sapudom et al., 2015). We determined the Young’s Modulus using the standard Hertz model fitted to the retract curve part.

### Invasion Assays in 3D Extracellular Matrices

For 3D invasion experiments, collagen monomer solutions were prepared as described above. Subsequently, 1.2 ml of the cooled collagen-buffer-solutions were added into each well of a 6-well plate and finally polymerized at 37°C and 95% humidity in an incubator for 2 h. The polymerized collagen scaffolds were rinsed 3 times using 2 ml Dulbecco’s phosphate buffered saline (PBS) per well. Afterward, 2 ml cell culture medium per well were added and each 6-well plate was incubated over night at 37°C, 95% humidity in an incubator (Fischer et al., 2017). Cells at a confluency of 80% were harvested using a 0.125% Trypsin/EDTA solution and 50.000 cells were seeded on top of the polymerized collagen networks in each well of a 6-well plate (Figure 2A, top). After incubation for 12 h at 37°C, 5% CO_2_, 95% humidity, a cell culture medium-drug solution was added and incubated for another 72 h at 37°C, 5% CO_2_, 95% humidity for cell invasion analysis (Figure 2A, bottom). Subsequently, the 3D invasion assays were fixed using 2.5% glutaraldehyde solution for 20 min in an incubator. After rinsing three times with PBS, a 4 μg/ml Hoechst 33342 dye solved in PBS was added and stored at 4°C overnight. These samples with stained nuclei were imaged using epifluorescence image-stacks recorded with a 20× objective (DMI8000B, Leica; Wetzlar, Germany), A4 filter cube (Leica), 0.55× c-mount adaptor (Leica) for the CCD camera (Orca-R2, Hamamatsu-Photonics, Munich, Germany). For each well of a 6-well plate, 121 fluorescence image stacks with a z-distance of 4 μm were recorded in a randomized 11 × 11 position grid. Nuclei positions are determined using a custom-built python application based on a sophisticated 3D image analysis and filter algorithm (Fischer et al., 2017). Cells adhered on top of the collagen matrix surface and located up to 8 μm below the surface are considered to be non-invasive, which accommodates minimal surface deviations for cell nuclei in the range of 15 μm (Kunschmann et al., 2017, 2019; Puder et al., 2019). Cells located deeper than 8 μm are considered to be invasive, since they can be clearly distinguished from the adherent cells on top of the collagen fiber matrix that represent the fraction of non-invasive cells. In addition, we modulated the invasive capacity of MDA-MB-231 and MCF-7 breast cancer cell lines by adding 20 μM of the matrix-metalloproteinase inhibitor GM6001 or a combination of GM6001 and 900 ng/ml TSA.

### Nuclear Size and Shape

To determine the shapes and sizes of the cell nuclei, we used the same invasion samples and data as described above for the 3D invasion assay. For the invasion assay, we have determined the exact positions of the cell nuclei. In the next step, we cut out the 3D image data around each individual cell nucleus, applied an image segmentation determining areas of cell nuclei and their surroundings. Subsequently, we determined shape parameters for each nucleus. We found that the minor axis length seems to be a good measure, as it accurately describes the nuclear shape while a cell potentially migrates through a collagen fiber pore (Figure 3A). When a cell must squeeze through a dense pore, it needs to deform its nucleus at its minor axis, whereas the major axis length is rather irrelevant. The decrease of the length of the minor axis is an indicator for how efficient cells have deformed their nuclei in order to squeeze through small collagen pores confinements.

### Actin Fiber Bundle Thickness and Mesh-Size

We fluorescently stained actin with 2 units ml^–1^ Alexa Fluor 546 Phalloidin dissolved in 1% BSA Hepes-buffer, the nucleus with 0.02 mg ml^–1^ Hoechst 33342 and the cell membrane with 0.25 mg ml^–1^ DiD for 4 h at 4°C. Fluorescent images were recorded using a confocal laser scanning microscope (Leica TCS SP8, Mannheim, Germany). Subsequently, we cut out regions of interest so that only actin fiber bundle network areas are cut out of the original confocal laser scanning microscopic images for analysis (Figure 3B, left). To determine the actin fiber bundle mesh-size and fiber thickness, we utilized a 2D implementation of our advanced pore size algorithm as described previously in Fischer et al. (2019). To determine the fiber thickness, we applied the algorithm on the actin phase of the segmentation (Figure 3B, right), similarly as for the collagen fibril thickness analysis of the collagen matrices in 3D (see above).

### Stiffness of Cell Nucleus and Cytoskeleton

To measure the stiffness of cells, we used a NanoWizard4 AFM system (JPK, Berlin, Germany). We used a cantilever with a 6 μm polystyrene bead glued to the tip (Figure 4A, top). In order to distinguish between cytoskeletal and nuclear stiffness, we probed each cell with two distinct measurement protocols. The cytoskeletons were probed utilizing a standard approach with 0.5 nN maximum indentation force at random measurement points along the perinuclear cytoskeleton (Fischer et al., 2017) (Figure 4A, bottom). The nuclei were probed at a single point centered above the nucleus and repeatedly measured 5 times with an indentation force of 5 nN (Krause et al., 2013) (Figure 4A, bottom). Exemplary force-distance curves for both nucleus and cytoskeleton are shown in Figures 4C,D, respectively. To distinguish the nucleus from other cell compartments, such as glycocalyx, plasma membrane and cytoskeleton, the nuclear force-distance curves were only analyzed at the topmost 10% of the curve (Figure 4C) (Krause et al., 2013). Both cytoskeleton and nucleus force-distance curves were analyzed using the standard Hertz model with the same processing- and fit-parameters using the JPK-SPM Data Processing v6.1.92 software. All fitted curves were independently filtered using the same parameters, such as a maximum residual root mean square (RMS) value of 150 pN. This procedure ensures that only curves were considered for evaluation that satisfy a certain goodness-of-fit criteria minimizing false values. RMS threshold values of ∼100 pN to ∼200 pN were sufficient for filtering out any force-distance curve containing artifacts, such as disturbed curves by dead cells or dirt particles passing the AFM cantilever, while preserving all other analyzable curves.

### Modulation of Nucleus Elasticity

In order to investigate the effect of nucleus stiffness on cell migration, the cells were treated with a 900 ng/ml trichostatin A (TSA) solution for 24 h. We tested concentrations between 150 and 1,200 ng/ml and found that this concentration is most effective for AFM analysis, while preserving the cell viability. In order to confirm that TSA affected the chromatin condensation using a computational approach as reported by Krotkov (1988) and Huang and Jing (2007), we analyzed the variations in the nuclear chromatin before and after TSA treatment (see Supplementary Figure S2). More precisely, for AFM measurements, each petri dish was treated with 900 ng/ml TSA 24 h before a measurement. For 3D migration assays, 900 ng/ml TSA were added after the cells adhered for 12 h on top of the collagen matrices. For control, we performed the TSA treatment (900 ng/ml) in the absence and presence of the matrix-metalloproteinase inhibitor GM6001 (20 μM) (see Supplementary Figure S3).

### Modulation of the Acto-Myosin Cytoskeleton

To reveal the role of the acto-myosin cytoskeleton on the cell stiffness, we altered the actin cytoskeleton pharmacologically. Firstly, we reduced the myosin II motor protein affinity to actin filaments in the actomyosin network using 25 μM Blebbistatin (Fischer et al., 2017; Kunschmann et al., 2017). Secondly, we used 0.2 μM Latrunculin A, which sequesters filamentous actin, resulting in monomeric actin inside the cells (Fischer et al., 2017; Kunschmann et al., 2017). Cells were treated with both pharmacological drugs 2 h prior to AFM measurement start.

### Statistical Analysis

All data are presented as mean or median values ± SD, as indicated in the figure legends. Since not all data were normal distributed, the statistical significance was determined using the Mann–Whitney *U*-test with standard significance levels of 5, 1, and 0.1%, which are indicated as ^∗^, ^∗∗^, and ^∗∗∗^, respectively.

## Data Availability Statement

The raw data supporting the conclusions of this article will be made available by the authors, without undue reservation, to any qualified researcher.

## Author Contributions

TF performed the majority of the experiments, analysis data, and wrote the manuscript. AH performed experiments and contributed to data analysis. CM designed the experiments, analyzed and interpreted the data, and wrote the manuscript. All authors reviewed the manuscript.

## Conflict of Interest

The authors declare that the research was conducted in the absence of any commercial or financial relationships that could be construed as a potential conflict of interest.
