# Supplementary material for: Effect of Nuclear Stiffness on Cell Mechanics and Migration of Human Breast Cancer Cells
Source: Front Cell Dev Biol. 2020 May 29;8:393. doi: 10.3389/fcell.2020.00393 (PMC7272586; doi:10.3389/fcell.2020.00393)
Supplement: Supplementary file 1 [file Data_Sheet_1.PDF]

Supplementary Material

Supplementary Figure 1

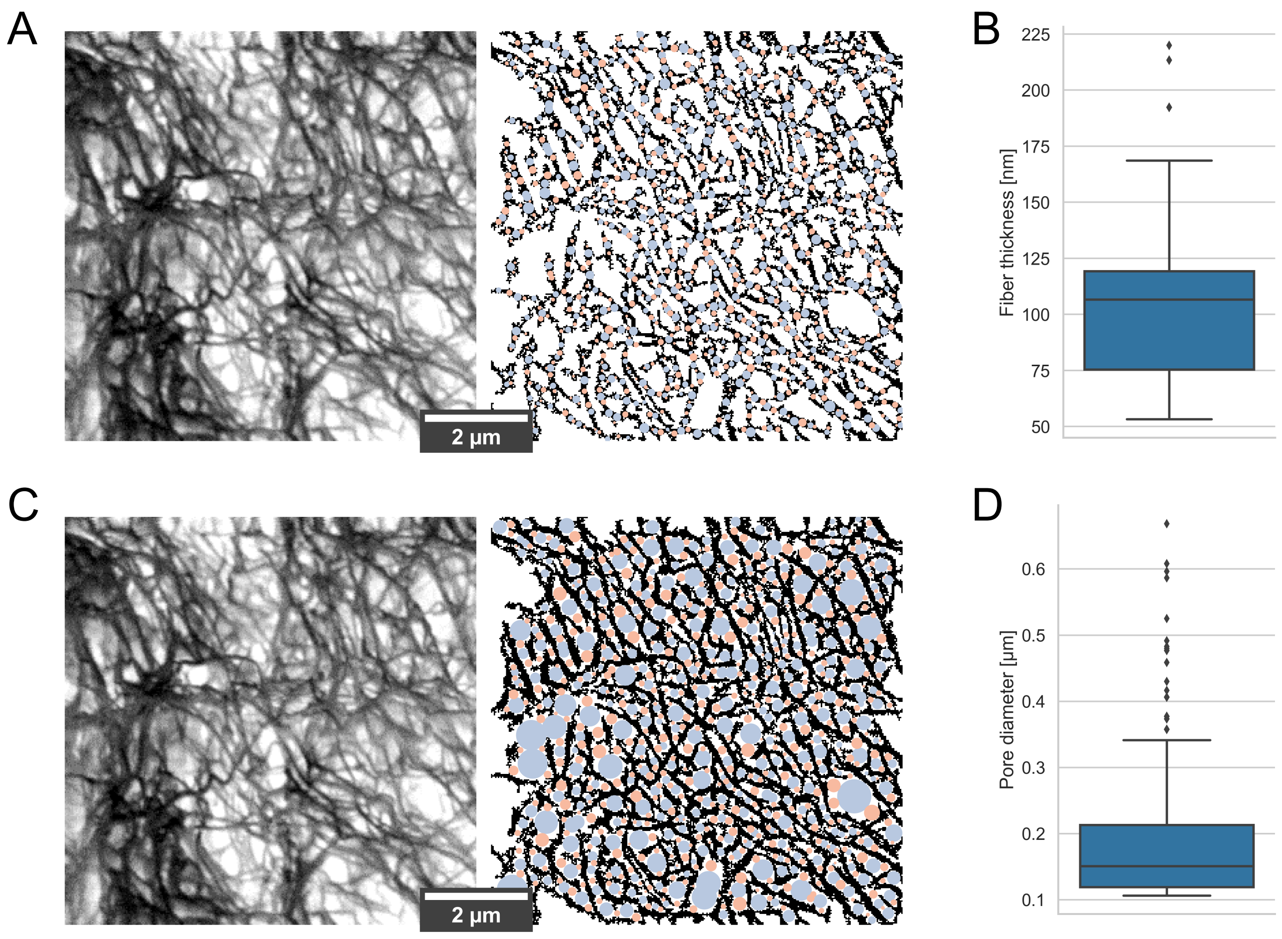

Supplementary Figure 1. Pore size and fiber thickness of a representative electron microscopic image (A and C) of a collagen fiber matrix revealed that the fiber thickness and the pore diameter are comparable to the scanning electron microscopic images (B and D). However, since it is known that the procedure of scanning electron microscopic images reduces the overall sample, the two parameters are decreased compared to the analysis of collagen matrices using laser scanning confocal images (see Figure 1).

Supplementary Figure 2

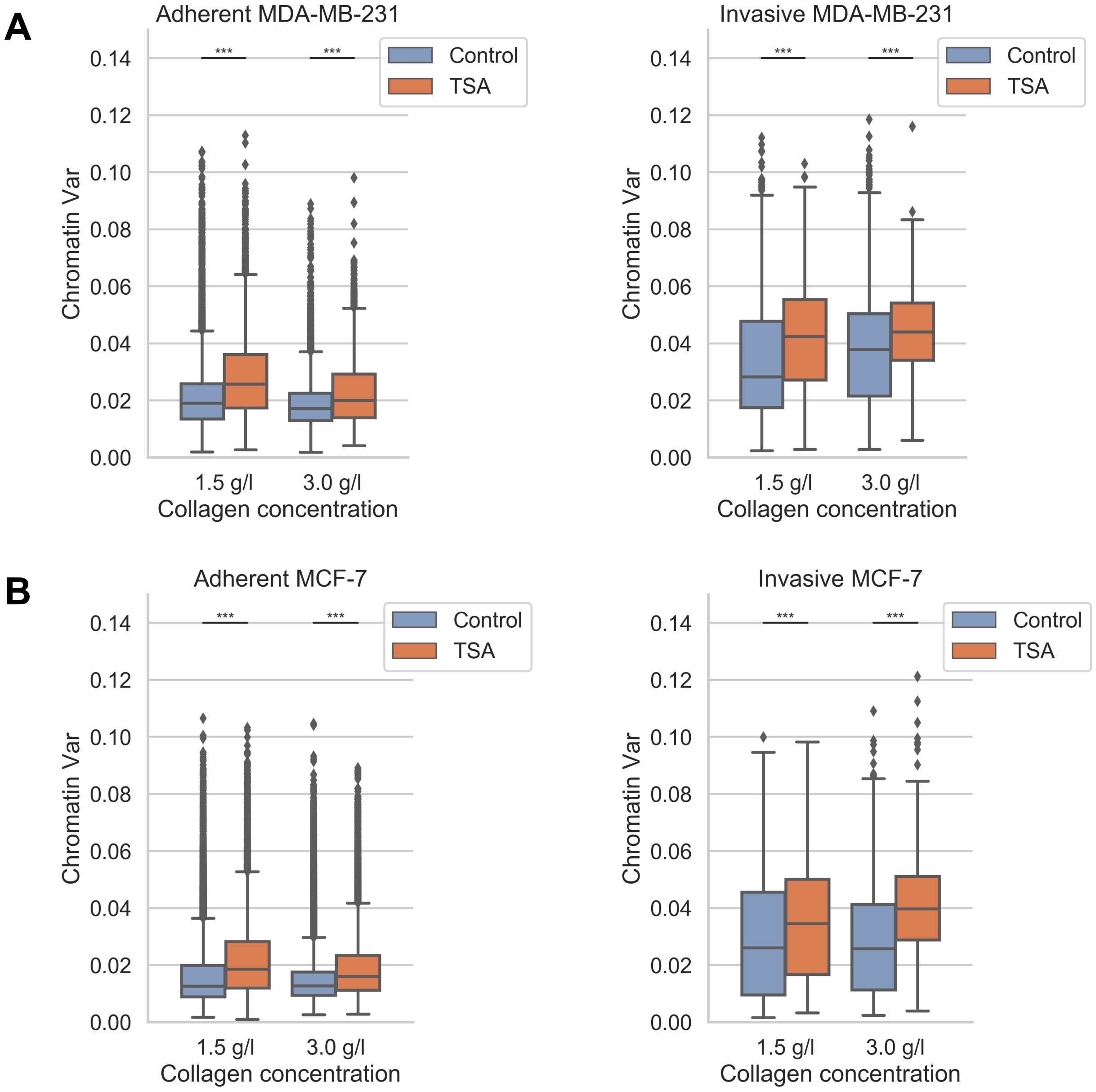

Supplementary Figure 2. Chromatin variation of adhesive and invasive MDA-MB-231 and MCF-7 cancer cells that have been seeded on top of 3D collagen fiber matrices and cultured for three days in the presence and absence of TSA. ***$p < 0.001$

Supplementary Figure 3

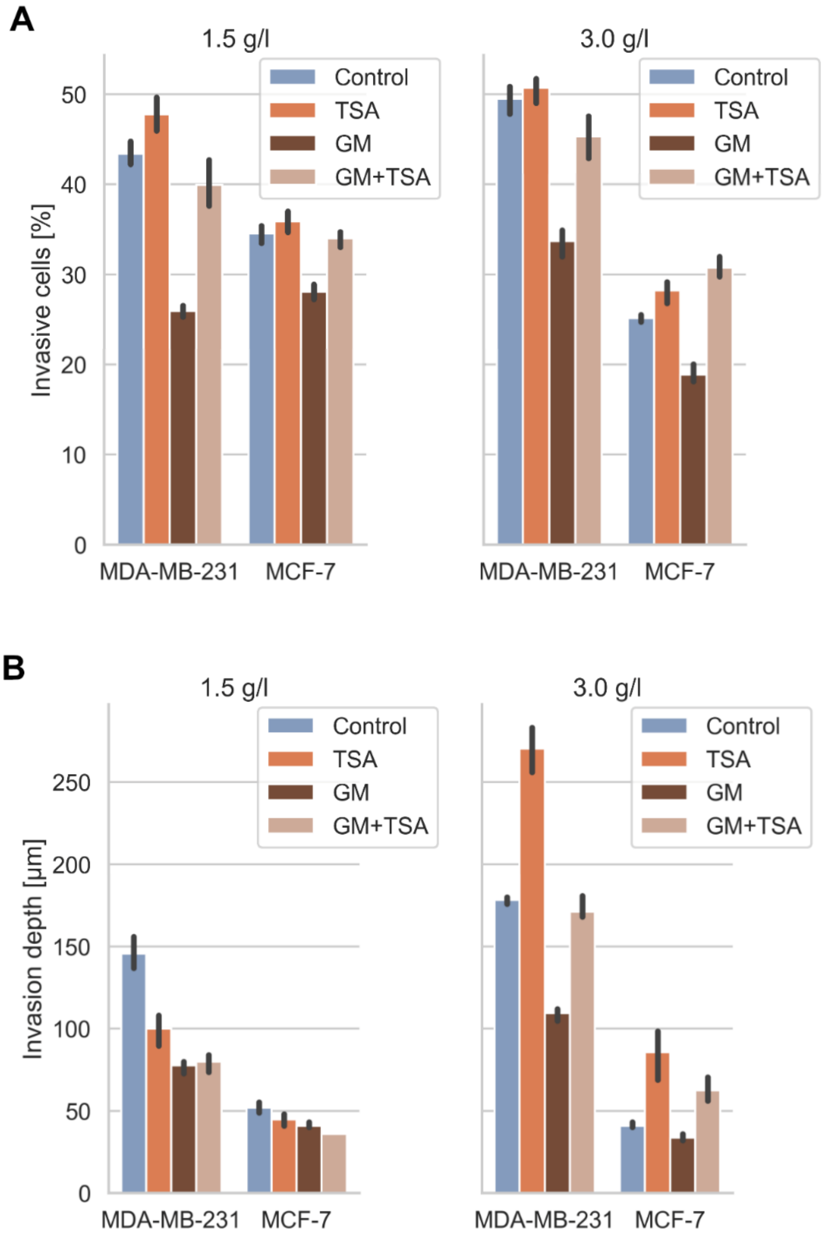

Supplementary Figure 3. The invasiveness of the human MDA-MB-231 and MCF-7 breast cancer cells in the presence and absence of the matrix-metalloproteinase inhibitor GM6001 (20 µM) or in the presence of GM6001 and 900 ng/ml TSA in both dense (3.0 g/L) and loose (1.5 g/L) 3D collagen matrices. A) The percentage of invasive cells MDA-MB-231 and MCF-7 cells is decreased after GM6001 treatment, whereas the effect of GM6001 can be rescued by addition of TSA. B) The invasion depths of MDA-MB-231 is pronouncedly decreased after GM6001 treatment, whereas the invasion depths of MCF-7 cells slightly decreased after GM6001 treatment.

Supplementary Figure 4

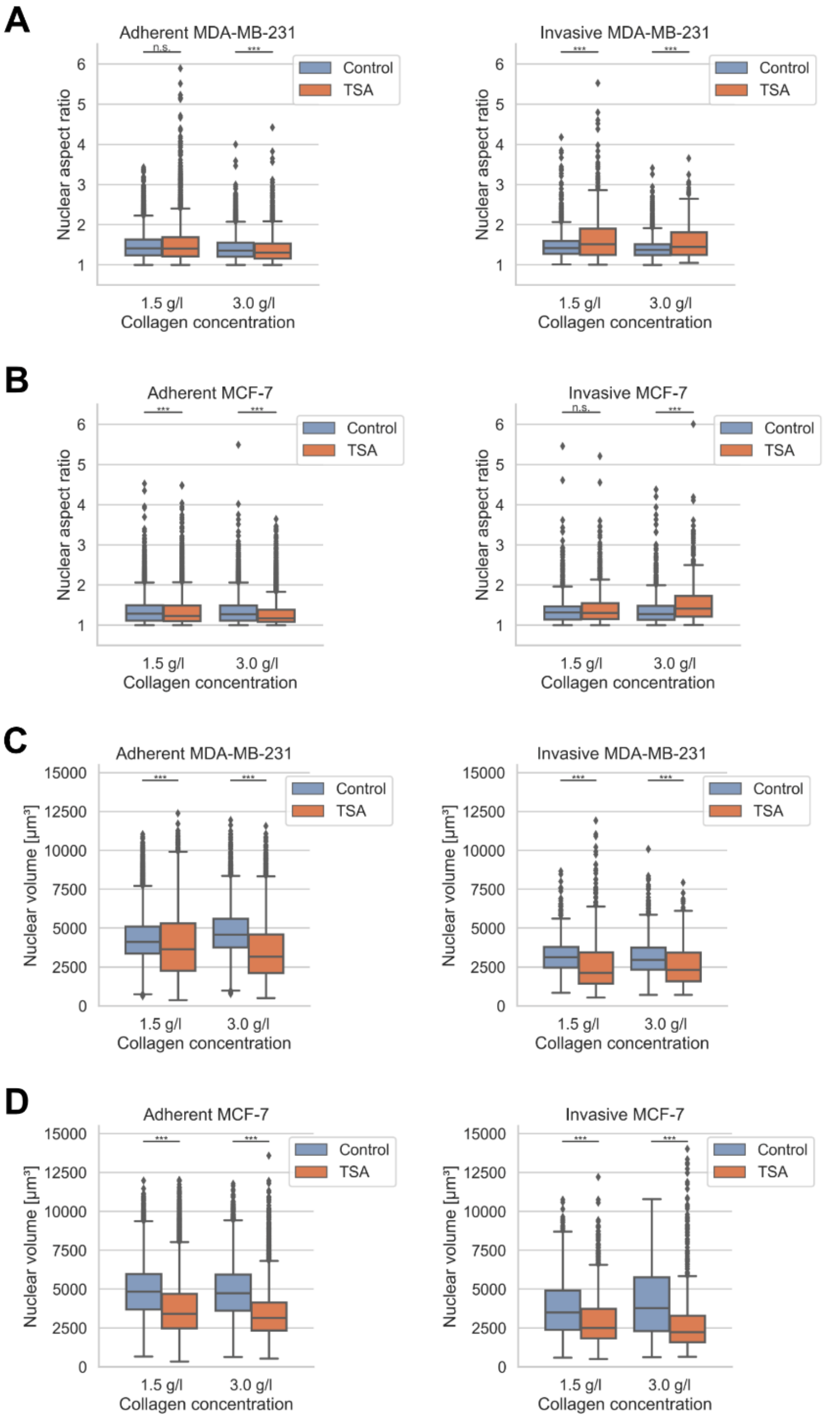

Supplementary Figure 4. Aspect ratio and nuclear volume of adhesive and invasive MDA-MB-231 and MCF-7 cancer cells that have been seeded on top of 3D collagen fiber matrices and cultured for three days in the presence or absence of TSA. n.s. not significant and ***$p < 0.001$

Supplementary Figure 5

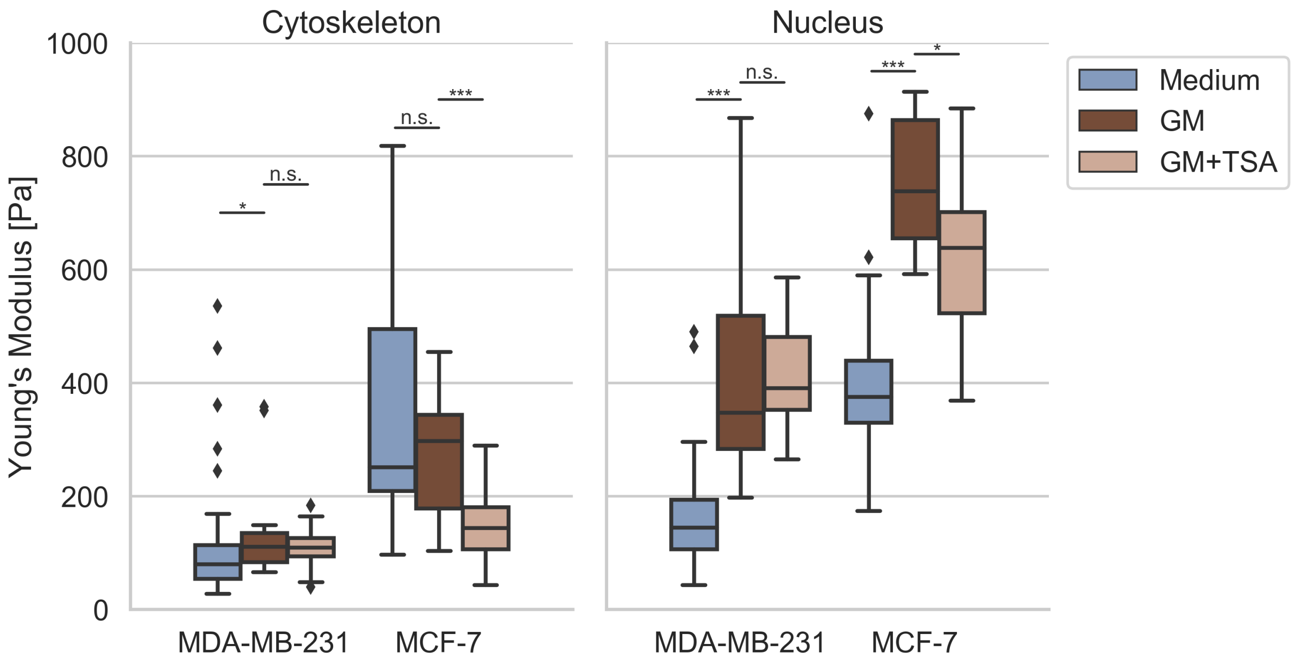

Supplementary Figure 5. Cytoskeletal and nuclear stiffness measurement of MDA-MB-231 and MCF-7 breast cancer cells using AFM in the presence and absence of the matrix-metalloproteinase inhibitor GM6001 (20 µM) or combined treatment with GM6001 and TSA (900 ng/ml). n.s. not significant, *$p < 0.05$ and ***$p < 0.001$

Supplementary Figure 6

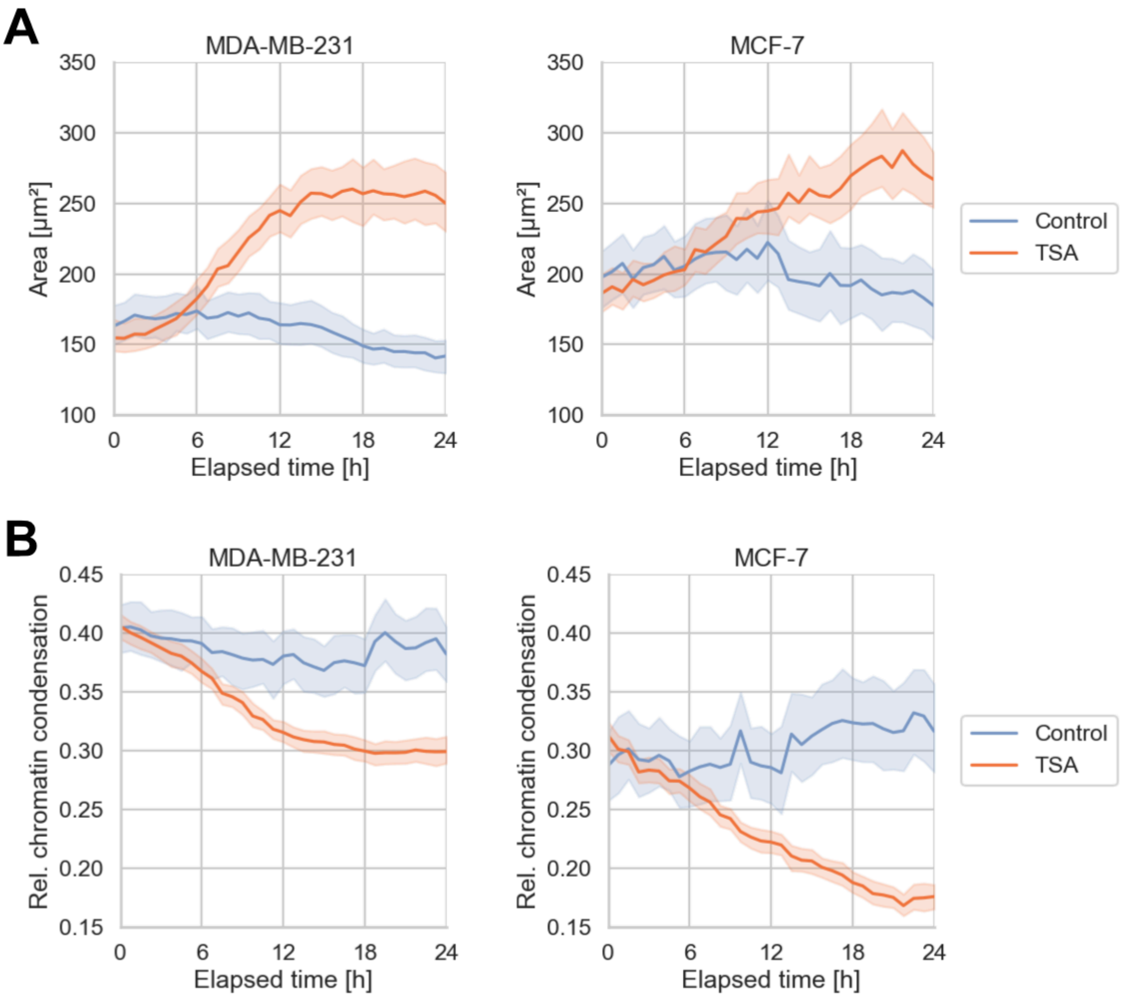

Supplementary Figure 6. Nucleus swelling and relative chromatin condensation of MDA-MB-231 and MCF-7 cells on 2D substrate. A) The nuclear area increased over time after TSA application, whereas the controls stayed unchanged. B) Under TSA treatment, chromatin de-condensation appeared. Controls showed no change in chromatin condensation. Analysis was performed using Cell Profiler v3.1.9 (McQuin et al., 2018).

Supplementary Figure 7

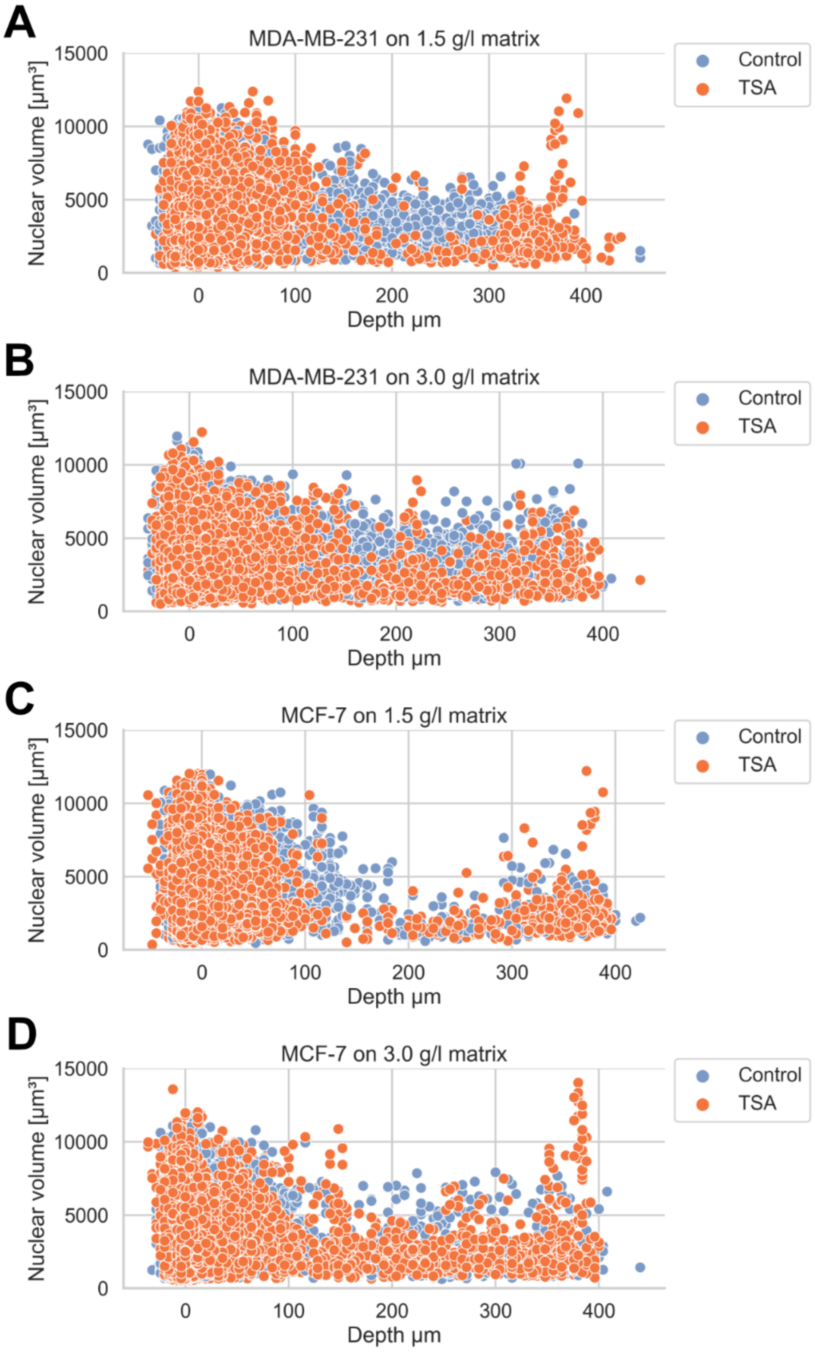

Supplementary Figure 7. Scatter plot of nuclear volume in dependence of penetration depth for both MDA-MB-231 and MCF-7 cell lines on loose and dense matrices under TSA treatment. No linear correlation between nuclear volume and penetration depth can be concluded. MDA-MB-231 cells adhered to and invaded in loose (1.5 g/l) collagen gels (A) and in dense (3.0 g/l) collagen gels (B) for three days. MCF-7 cells adhered to and invaded in loose (1.5 g/l) collagen gels (C) and in dense (3.0 g/l) collagen gels (D) for three days.

Supplementary Figure 8

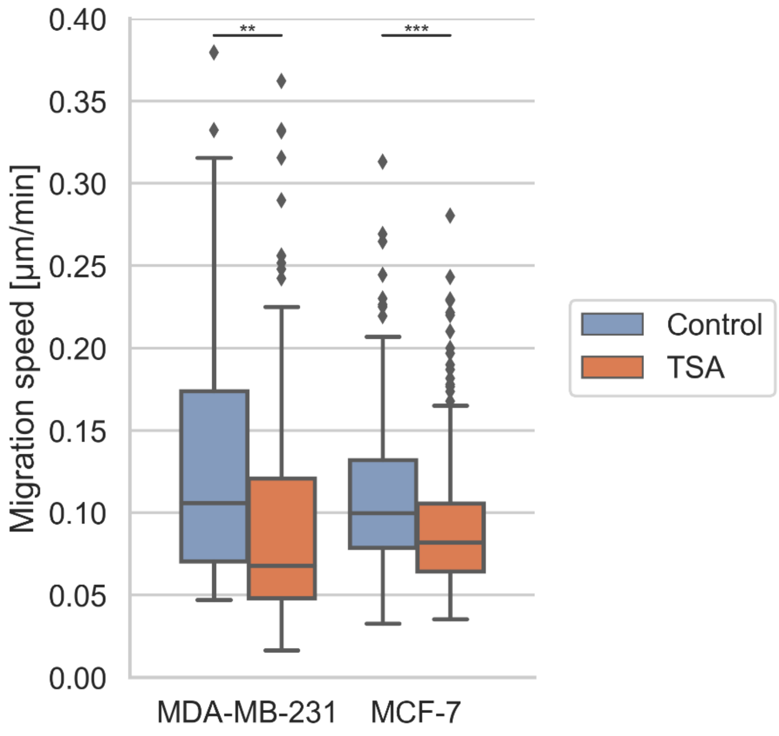

Supplementary Figure 8. Migration speed of MDA-MB-231 and MCF-7 cells under TSA treatment and controls on 2D plastic substrate absent of any steric hindrances.

References

McQuin, C., Goodman, A., Chernyshev, V., Kamentsky, L., Cimini, B. A., Karhohs, K. W., et al. (2018). CellProfiler 3.0: Next-generation image processing for biology. *PLOS Biol.* 16, e2005970. doi:10.1371/journal.pbio.2005970.
